# Implementation Science to Increase Adoption of Genomic Medicine: An Urgent Need

**DOI:** 10.3390/jpm11010019

**Published:** 2020-12-29

**Authors:** Hana Bangash, Iftikhar J. Kullo

**Affiliations:** Department of Cardiovascular Medicine, Mayo Clinic, Rochester, MN 55905, USA; bangash.hana@mayo.edu

Advances in genomics have the potential to improve human health [1] and, increasingly, healthcare providers (HCPs) are expected to incorporate genomic data in clinical decision making [2]. However, adoption of genomic medicine in clinical practice remains a work in progress [3,4]. HCPs support such adoption [5], but may be constrained by several factors including lack of genomics knowledge and training, a paucity of electronic health record (EHR)-based digital tools for genomics, and the burden of EHR documentation [6,7]. To identify potential barriers to effective genomic medicine integration in diverse settings, Obeng et al. surveyed 285 physicians at the Implementing GeNomics In pracTicE (IGNITE) sites [3].

Established in 2013 by the National Institutes of Health, the IGNITE Network aims to promote widespread adoption of genomic medicine in clinical practice and to establish and disseminate best practices related to such implementation [8]. The Network comprises six participating sites, each of which is conducting genomic medicine studies, such as integrating pharmacogenomics (PGx) data in clinical care, refining disease risk prediction, and developing point of care tools for education and clinical decision making [8]. Obeng et al. surveyed physicians from five of the six IGNITE sites over the course of two years. Physician responses were compared based on: (1) the type of study being conducted at the respondent’s site: pharmacogenomics or disease genetics, and (2) physician characteristics. Using constructs from the Consolidated Framework for Implementation Research (CFIR), which were modified by the IGNITE Common Measures Workgroup, the authors designed a survey that was completed by physicians either in-person or electronically [3].

Two-thirds of respondents considered PGx or genetic testing as relevant to their clinical work and one-third believed that access to genetic risk information has potential to improve their ability to care for patients. However, a majority of physicians felt that their training had not prepared them to work with patients at increased genetic risk, with only 15% of physicians indicating confidence in being able to apply genetic test results in clinical care [3]. Physicians also lacked awareness of the availability of resources that could be used to convey genetic information. Respondents from PGx sites were more likely than those from disease genetic testing sites to identify genetic testing as being useful, feel prepared and confident in applying genetic test results in clinical care, and be aware of available resources, indicating an overall greater acceptance of PGx testing. The authors postulated that the increased acceptance of PGx testing may have been due to existence of specific and actionable PGx test results, strong supporting evidence, established guidelines for use, and potential for testing to improve health outcomes in patients, with implications for a large proportion of the population [3]. These observations are relevant for the adoption of non-PGx genetic tests and highlight the need for using implementation science to facilitate adoption of genomic medicine in clinical settings.

The National Human Genome Research Institutes’ strategic vision for the next decade includes a central role for implementation science to increase uptake of evidence-based genomic interventions in clinical practice [4]. Research findings take an average of 17 years to move from discovery to translation in healthcare settings—a lost opportunity for improving health outcomes [9,10]. A systematic review of 283 genomic medicine studies found that only 1.8% used implementation frameworks, largely in early research stages with lack of data on adoption and sustainability of interventions [2]. These results highlight the urgent need to use implementation science during all phases of research and to assess multilevel contextual variables that could impact genomic medicine integration, both at the institutional and HCP levels. Frameworks such as CFIR, RE-AIM (Reach, Effectiveness, Adoption, Implementation and Maintenance), and outcome measures can be applied during research, development, and integration of genomic interventions to identify and overcome barriers, decrease time to translation, ensure effective adoption, promote HCP satisfaction, and improve health outcomes [11,12,13]. Additional evaluation and adaptation can be carried out to ensure that implementation is successful and sustainable.

Institutions and health systems need to develop training and education modules to enhance the genomics competencies of HCPs and integrate genomic clinical decision support (CDS) and additional digital tools in the EHR [14]. Digital tools lie at the intersection of genomic medicine and health information technology and provide guidance to HCPs related to genomic medicine and include CDS, decision aids, web and smartphone applications (apps), and chatbots. Although HCPs are the main end users, often they are not included in the process of development and integration of these tools. In a study to develop a CDS tool for familial hypercholesterolemia, a prevalent and monogenic disorder, input was sought from HCPs on CDS content, layout, and clinical integration. The study findings illustrated some of the challenges that are encountered during genomic medicine implementation: HCPs were unable to distinguish genetic from non-genetic hypercholesterolemia, they lacked confidence in ordering appropriate genetic testing for patients, and experienced difficulties in locating genomic resources such as relevant order sets, decision aids, and patient education materials in the EHR [7]. These results highlight the value of engaging with stakeholders such as HCPs early in the process of digital tool development to identify barriers. Qualitative interviews, focus groups, surveys, and user experience testing can be used to engage with HCPs, enabling them to share their perspectives on tool prototypes. Input from HCPs can subsequently inform iterative refinements of tools to ensure they appropriately fit the real-world settings. HCP feedback can also be used to better understand diverse clinical workflows and proactively identify bottlenecks/hurdles prior to large-scale implementation.

HCPs should be educated about such resources, including where to locate them, the scenarios in which to use them, and how to integrate them into clinical workflows without increasing cognitive burden. An intelligent EHR could display relevant tools and auto-populate them with pertinent information during clinical encounters so that HCPs can spend more time interacting with patients rather than with the EHR [15]. Grand rounds, departmental meetings, and electronic newsletters, along with specific education modules and interactive sessions, are some of the strategies that can be used to promote knowledge dissemination among HCPs [7,16]. Providing easily accessible technical support is also likely to increase tool use and enhance HCP satisfaction. Institutions and health systems should identify physician champions who can facilitate genomic medicine implementation by serving as early adopters and opinion influencers.

Much work remains to be conducted to increase adoption of genomic medicine in clinical practice. Integration of genomic data and evidence-based interventions in clinical workflows has the potential to improve public health and reduce health disparities by enabling broader access to resources. Obeng et al. illustrated that although providers value genomic medicine integration in practice, they are unable to fully use genomic data due to limited resources and a perceived lack of knowledge and skills. Applying implementation science principles to genomic medicine research, engaging with HCPs during the development and deployment of digital tools, and increasing genomics-based education and training of HCPs can promote awareness and uptake of genomic medicine in clinical practice and further the goal of precision medicine.
